# An Explainable Multimodal Neural Network Architecture for Predicting Epilepsy Comorbidities Based on Administrative Claims Data

**DOI:** 10.3389/frai.2021.610197

**Published:** 2021-05-21

**Authors:** Thomas Linden, Johann De Jong, Chao Lu, Victor Kiri, Kathrin Haeffs, Holger Fröhlich

**Affiliations:** ^1^ Department of Bioinformatics, Fraunhofer Institute for Algorithms and Scientific Computing (SCAI), Schloss Birlinghoven, Sankt Augustin, Germany; ^2^ Bonn-Aachen International Center for Information Technology (B-IT), University of Bonn, Bonn, Germany; ^3^ UCB Biosciences GmbH, Monheim, Germany; ^4^ UCB Ltd., Raleigh, NC, United States

**Keywords:** precision medicine, personalized medicine, P4 medicine, comorbidity prediction, administrative claims data, epilepsy, neural networks, machine learning

## Abstract

Epilepsy is a complex brain disorder characterized by repetitive seizure events. Epilepsy patients often suffer from various and severe physical and psychological comorbidities (e.g., anxiety, migraine, and stroke). While general comorbidity prevalences and incidences can be estimated from epidemiological data, such an approach does not take into account that actual patient-specific risks can depend on various individual factors, including medication. This motivates to develop a machine learning approach for predicting risks of future comorbidities for individual epilepsy patients. In this work, we use inpatient and outpatient administrative health claims data of around 19,500 U.S. epilepsy patients. We suggest a dedicated multimodal neural network architecture (Deep personalized LOngitudinal convolutional RIsk model—DeepLORI) to predict the time-dependent risk of six common comorbidities of epilepsy patients. We demonstrate superior performance of DeepLORI in a comparison with several existing methods. Moreover, we show that DeepLORI-based predictions can be interpreted on the level of individual patients. Using a game theoretic approach, we identify relevant features in DeepLORI models and demonstrate that model predictions are explainable in light of existing knowledge about the disease. Finally, we validate the model on independent data from around 97,000 patients, showing good generalization and stable prediction performance over time.

## Introduction

Epilepsy is a complex, life-threatening brain disorder characterized by repetitive seizure events. Epilepsy patients often suffer from various and severe physical and psychological comorbidities, such as overweight and obesity, anxiety, migraine, bipolar disorder, and cardiovascular diseases (Seidenberg et al., 2009; Ottman et al., 2011; Keezer et al., 2016). Some comorbidities confer a poor disease prognosis because they complicate pharmacological treatment owing to possible drug–drug interactions and adverse events (Verrotti and Mazzocchetti, 2016). The actual development of comorbidities is dependent on patient-specific factors and may be modulated by antiepileptic drug (AED) treatment (Zaccara, 2009). Early identification and treatment of comorbidities has thus been identified as highly relevant to improve the quality of life of epilepsy patients (Verrotti and Mazzocchetti, 2016). However, there is a high subject-to-subject variability. Methods from the field of artificial intelligence (AI), and more specifically machine learning (ML), have the potential to predict comorbidity risks on an individual subject basis, hence fulfilling one of the promises of a more individualized patient care in the sense of precision medicine. More specifically, ML-based approaches can be used to aid disease prevention by predicting the time-dependent risk of an individual epilepsy patient to develop several common comorbidities in the future, such as 1) anxiety, 2) bipolar disorder and schizophrenia, 3) diabetes type 2, 4) migraine, 5) overweight and obesity, and 6) stroke and ischemic attacks.

Machine learning models to predict individualized comorbidity risks of diseases different from epilepsy have recently been published (e.g., Dworzynski et al. (2020) and Noh et al. (2020)) using clinical routine data from the Danish national registry and hospital electronic health records, respectively. For epilepsy, Glauser et al. (2020) proposed an ML model for psychiatric comorbidities based on survey data from 122 patients. In our earlier work (Gerlach et al., 2017), we proposed an ML model (random survival forests) using U.S. administrative health claims data from ∼10,000 epilepsy patients to predict several major comorbidities (anxiety, bipolar disorder and schizophrenia, diabetes type 2, migraine, overweight and obesity, and stroke and ischemic attacks) of epilepsy patients.

Administrative health claims data have generally been shown useful for developing ML models in the epilepsy field. For example, An et al. (2018) used claims data of more than 1.3 million epilepsy patients to predict antiepileptic drug resistance. Examples from other disease areas include prediction of Alzheimer’s disease (Park et al., 2020), osteoporotic hip fractures (Engels et al., 2020), and heart failure (Desai et al., 2020). The opportunities of healthcare claims data for ML-based modeling have further been discussed in (Fröhlich et al., 2018; Miotto et al., 2018; Xiao et al, 2018; Thesmar et al., 2019; Kwak and Hui, 2020).

In our earlier work, we demonstrated the possibility to augment claims data with biomedical background knowledge, hence enabling the interpretation of machine learning models down to the level of disease-associated biological processes (Gerlach et al., 2017). The particular novelty of the present work is a dedicated multimodal neural network architecture for administrative claims data, which we call Deep personalized LOngitudinal convolutional RIsk model (DeepLORI). We show that DeepLORI more accurately predicts the time-dependent risk for six common comorbidities on the level of individual patients than several competing methods, including our own previously proposed model. Using a game theoretic approach based on Shapley Additive Explanations (Lundberg and Lee, 2017), we show that DeepLORI models are explainable, also on the level of predictions for individual patients.

## Data

### Claims-Based Electronic Health Records

U.S. commercial inpatient and outpatient data were obtained from IBM® MarketScan® Truven Health databases. The Commercial Claims and Encounters database within MarketScan® is a nationally representative collection of de-identified patient-specific inpatient, outpatient, and pharmaceutical claims from more than 200 insurance carriers and large, self-insuring companies. All dates and time stamps were transformed from a daily to a monthly scale (1 month = 30 days) for a more robust representation. The data generally comprise demographic (age, gender) and regional information (major metropolitan area), days in hospital, health insurance plan, and time-dependent diagnosis codes and prescriptions (plus prescription duration and quantity).

We used two cohorts: 1) the original data covering years 2011–2015 for model training and evaluation within a nested cross-validation scheme, and 2) the external validation data covering years 2008–2018 to validate the models trained on the “original data.” In agreement to our earlier publication (Gerlach et al., 2017) and common practice at UCB, epilepsy patients in the original data were identified matching at least one of the following criteria:

(1) An occurrence of ≥ 2 ICD-9-CM codes of 345.xx (i.e., epilepsy, except 345.3—grand mal status) among separate medical encounters (separate dates in any care venue)
(2) An occurrence of ≥1 ICD-9-CM code of 345.xx (except for 345.3) AND ≥1 ICD-9-CM code of 780.39 (convulsions) among separate medical encounters
(3) An occurrence of 1 ICD-9-CM code of 345.xx (except for 345.3) and code(s) for AED prescription at least a day after the 345.xx code
(4) An occurrence of ≥2 ICD codes of 780.39 among separate medical encounters and code(s) for AED treatment. The code(s) for the AED treatment should occur at least a day after the second 780.39 irrespective of the presence or absence of an AED code after the first 780.39 code
(5) Individuals with ICD-9-CM code 345.3 will be required to have an occurrence of ≥2 ICD-9-CM codes of 345.3 separated by at least 30 days, or an occurrence of the 345.3 code and ≥1 ICD-9-CM code 780.39 separated by at least 30 days, or ≥1 ICD-9-CM code 345.3 and ≥1 ICD-9-CM code 345.xx encounters on separate days

The index date for each patient was defined as the time point of the first epilepsy diagnosis, and for definitions requiring at least 2 ICD-9-CM codes, the first diagnosis code was the index date. The data were further filtered by requiring for each patient 1) at least 365°days of medical history before, and 365-day follow-up after the index date; 2) age between 18 and 65 years; 3) any AED treatment during the observation period. Altogether this yielded 7,430,840 records from 19,510 patients. More details about the filtering process can be found in the Supplementary Material of this article. For part of these patients, diagnoses after the index date were coded in ICD10, which we mapped to ICD-9-CM via the Thomas Reuters^TM^ public Web resource^1^ and manual curation.

Note that in medical practice, confirmation of the final diagnosis “epilepsy” can be complicated and often requires a number of visits. Moreover, reporting of a dedicated diagnosis within our data does not necessarily correspond to the actual time point of the medical condition within the patient. To capture this uncertainty, we defined a three-month time interval starting from the index date as the “epilepsy diagnosis period”. That means the actual medical history of each patient after application of the abovementioned filter criteria was 365 + 91°days, that is, 456°days.

Diagnosis codes after 1st Oct 2015 were provided as ICD-10-CM codes. Accordingly, the following modified inclusion criteria were applied to select epilepsy patients in the external validation data (covering 2008–2018):

(1) The presence of at least 1 ICD-9-CM of 345.xx or ICD-10-CM of G40.xx (epilepsy)
(2) The presence of at least 2 ICD-9-CM of 780.39 or ICD-10-CM of R56.9 (convulsions) within one year.

After applying the same filter criteria as for the original data, this resulted in 112,755 patients. Within those patients, we pre-filtered diagnosis codes and substances observed in ≤10 patients or with a frequency ≤1%. One of the main issues with claims data is that one and the same diagnosis may be coded with different ICD codes. Moreover, observations related to one specific ICD9/10 code could be rare. To address these issues, we mapped all ICD-9-CM codes to PheWAS terms, which describe a higher level aggregate of several ICD codes (Carroll et al., 2014). In addition, a mapping to MeSH (Rogers, 1963) was performed to allow for integration with other data sources (see *Methods*).

### Definition of Focused Comorbidities and Compilation of Training Data

Based on the medical literature and observed frequency in our data, we focused on six common comorbidities of epilepsy patients: 1) anxiety, 2) bipolar and schizophrenia, 3) diabetes type 2, 4) migraine, 5) overweight and obesity, and 6) stroke and ischemic attack. These comorbidities were defined according to a set of PheWAS codes provided in the supplements (Supplementary Table S1).

The number of patients with these comorbidities being diagnosed at least 6 months after the epilepsy diagnosis period differs widely across comorbidities (Table 1). We would like to highlight that our data are in principle right-censored, that is, the diagnosis of a specific comorbidity might happen after the end of the period covered by our training data. Moreover, a significant proportion of those individuals where a diagnosis of a specific comorbidity is observed (i.e., incident cases) have already been diagnosed with at least one of the other 5 comorbidities during their medical history (Table 1). Note that for training a machine learning model to predict a specific comorbidity, we should not have an observation of the same comorbidity in the medical history of any of the training samples. For this reason, the number of patients in the training data is different per comorbidity, and we developed separate machine learning models for each comorbidity.

**TABLE 1:** Proportion of incident patients by comorbidity.

Comorbidity	*n* total	*n* incident	% Incident	Mean ± sd, no. of other comorbidities in medical history per incident patient
Anxiety	15,274	4,037	26%	1.3 ± 0.6
Bipolar/Schizophrenia	16,908	1,279	8%	1.5 ± 0.8
Diabetes type 2	16,929	1,377	8%	1.5 ± 0.8
Migraine	16,036	2,063	13%	1.3 ± 0.7
Overweight/Obesity	17,273	3,307	19%	1.4 ± 0.7
Stroke/Ischemic Attack	17,208	1,157	7%	1.5 ± 0.8

Each diagnosis and prescription in our data has an associated time stamp. Due to the fact that the appearance of a record in our data does not necessarily correspond to the observation of the actual medical condition, each time stamp was mapped to a monthly (= 30-day time interval) resolution.

## Methods

### Proposed Model: DeepLORI

#### DeepLORI Architecture

As highlighted before, our aim was to develop separate machine learning models for each of six typical comorbidities of epilepsy patients. Each of these models aims for predicting the time-dependent risk of an individual to be diagnosed with one specific comorbidity.

We came up with a dedicated neural network model for our purposes, which we call Deep personalized LOngitudinal convolution RIsk model (DeepLORI). We start by explaining the principle architecture of DeepLORI. In agreement to our former work, one of the key ideas is that claims data have an inherent hierarchical structure (Gerlach et al., 2017): The data initially contain three major types of features: 1) prescribed substance codes, 2) diagnoses codes (mapped to PheWAS terms, see above), and 3) general demographic information, such as age, gender, and major metropolitan area information. Monthly reported prescriptions and diagnoses can typically be represented via a one-hot vector encoding. However, individual substance and diagnose codes are typically, rather sparsely, observed over time, which can potentially lead to challenges for a machine learning algorithm to find regularities.

**FIGURE 1:**
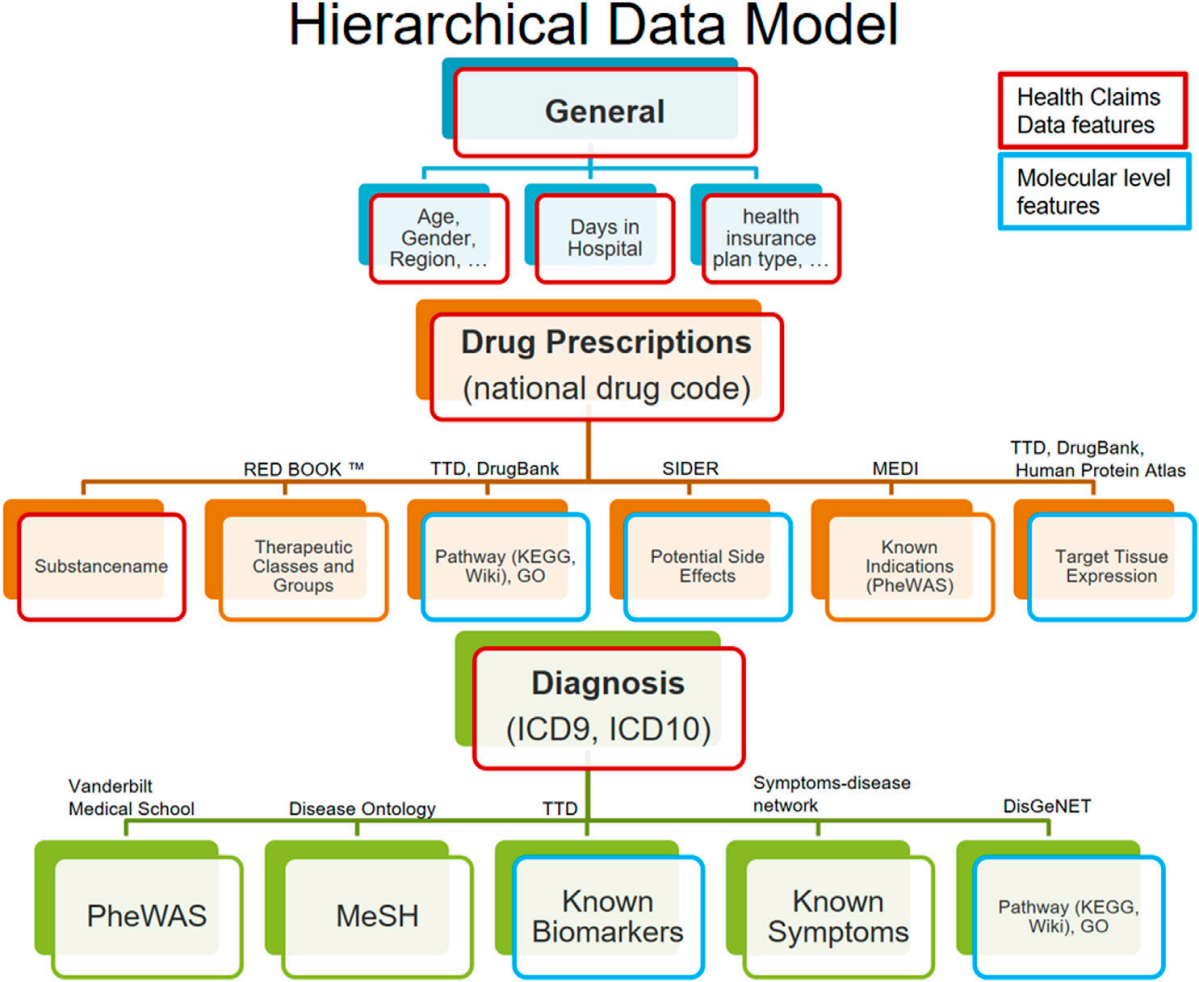
Hierarchical data model structures the data in 3 input domains (general, drug, and diagnosis) and according subdomains. Features retrieved from the claims data are highlighted in red, and all others are augmented features (including those highlighted in blue).

Based on this consideration, our idea was to use additional background knowledge available in databases to impose further hierarchical substructure: For example, each prescribed substance may have one or several known targets, and it can have a number of side effects reported in clinical studies. Diagnoses have associated symptoms, and in some cases, biomarkers may exist. Based on this background information, each domain of features (e.g., diagnosis) can be further associated to several subdomains (e.g., biomarkers and impaired biological pathways). Subdomain features can subsequently be represented via a one-hot vector encoding. Figure 1; Table 2 provide an overview about the domains and corresponding subdomains we defined in our data. (More details about our previously published approach to augment claims data with biomedical knowledge can be found in the supplements.)

**TABLE 2:** Number of features by domain, subdomain, and feature origin (claims data or biomedical background knowledge).

Domain	Subdomain	Feature origin		#Time-dependent features (per 30-day time interval)	#static features
		Claims data	Background knowledge		
Diagnosis	Biomarker		X	699	
Diagnosis	GO term		X	3,759	
Diagnosis	MeSH disease		X	137	
Diagnosis	Pathway		X	453	
**Diagnosis**	**PheWAS**	x		**1,196**	
Diagnosis	Symptoms		X	235	
**General**	**Age, sex, insurance, hospital**	x			**17**
General	Medical risk		X		53
**General**	**Region**	x			**403**
Drug	GO term		X	1,240	
Drug	Pathway		X	435	
Drug	PheWAS (known indications)		X	986	
Drug	Side effects x frequency		x	1,489	
Drug	**Substance name**	x		**453**	
Drug	Target tissue		x	32	
Drug	Therapeutic class		x	104	
Drug	Therapeutic group		x	19	
				∑=11,237	∑=437

Bold values indicate that the “Feature origin” is “Claims Data”.

**FIGURE 2:**
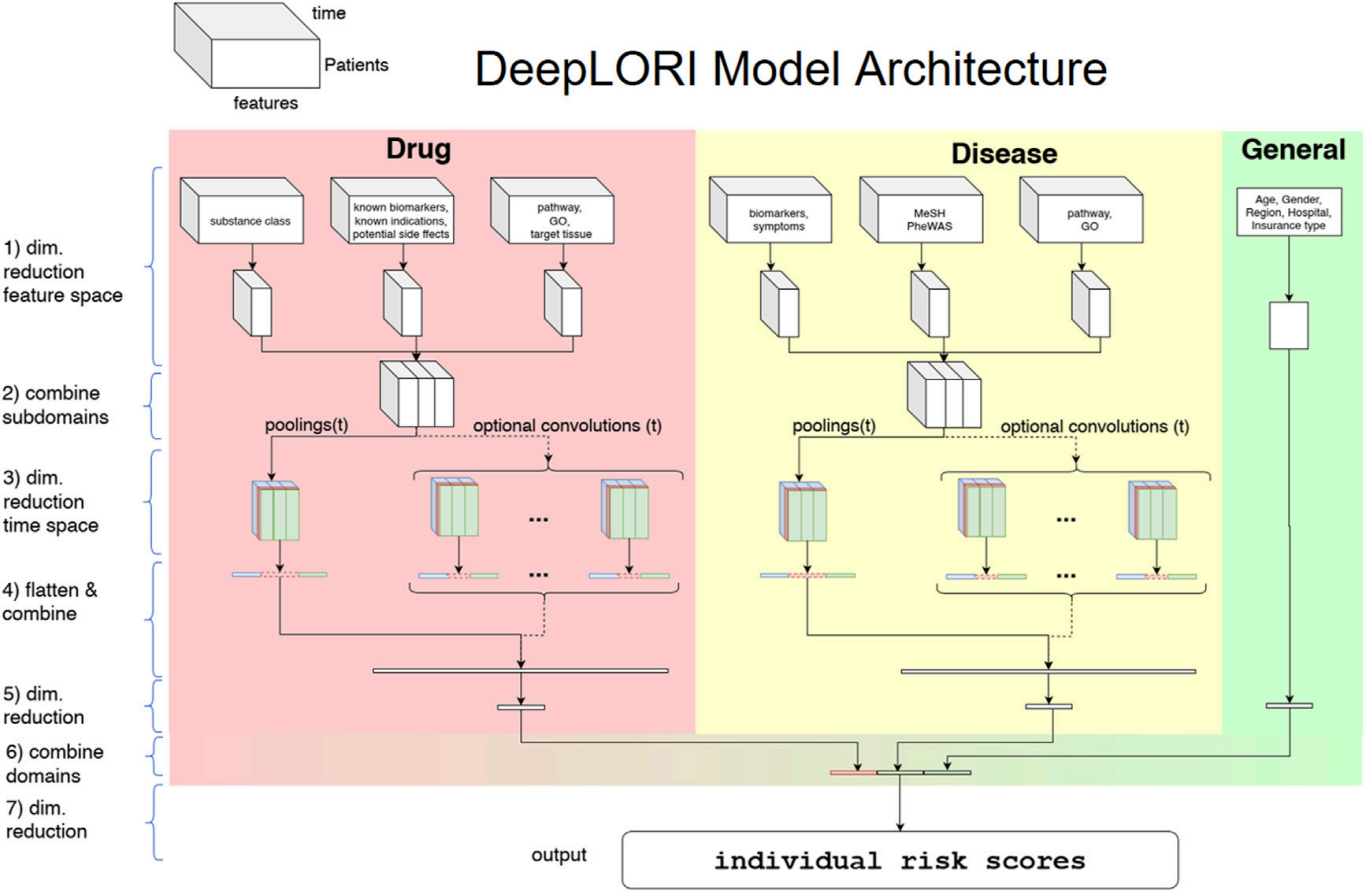
DeepLORI model architecture: The input is organized as a multimodal data cube, which is subdivided into several feature domains. Medication and diagnosis-related features are time-dependent, whereas features in the “general” domain are not. The three different colors at stage (3) symbolize the 3 different pooling/convolution kernel sizes.

In this work, we propose a multimodal neural network architecture to reflect the specific structure of the augmented claims data (see Figure 2). In this architecture, each of the feature domains and subdomains are initially treated as separate data modalities. Note that each feature derived from diagnosis and substance codes has an additional time stamp (30-day interval), that is, each subdomain is a three-dimensional data cube. Each of these tensors is projected down to a lower dimensional representation via bottleneck feedforward architecture with 1–4 hidden layers, where the exact number of hidden layers and units per layer are treated as tunable hyperparameters in our framework (see details in Supplementary Material). In conclusion, at the first layers for each subdomain, specific latent features are extracted in a nonlinear manner from the original data and subsequently concatenated.

After latent feature extraction, DeepLORI models the time-dependency in the data within each feature domain. For that purpose, we apply a pooling function (max or mean) over the entire time series simultaneously for all feature domains, but individually for each component of our one-hot vector representations. The exact choice of the pooling function is a hyperparameter. In addition to pooling, we allow for the application of different time convolutional kernels (with multiple filters per kernel), similar to a sliding window. Whether or not convolutional filters are applied and which sizes these filters have are again determined within hyperparameter optimization.

After modeling time-dependency, there is another feedforward bottleneck structure (same tunable design as for the initial latent feature extraction) in our network. Finally, DeepLORI concatenates latent features extracted from each feature domain and feeds them through the last feedforward bottleneck structure into one output unit, representing a patient-specific comorbidity risk score. That means we have one DeepLORI model per comorbidity. (A more detailed view on the DeepLORI architecture, including an overview of all tunable hyperparameters can be found in Supplementary Material.)

#### Loss Function

Let the training data be denoted as $D=\left\{\left(x_{it},y_{i},\delta_{i}\right)|\;x_{it}\in {\mathbb{R}}^{d\times T},y_{i}\in \mathbb{R},\delta_{i}\in \left\{0,1\right\},\;i=1,2,\ldots ,n,\;\;t=1,2,\ldots ,T\right\}$, where T is the maximum number of time stamps in the data (in our case, 15 months) and $y_{i}$ is the observed time of the first diagnosis of a comorbidity (in case data are not censored) after the index date or the maximum observed event free time. Moreover, $\delta_{i}\in \left\{0,1\right\}$ is a binary variable indicating whether $y_{i}$ is right-censored $\left(\delta_{i}=0\right)$ or not $\left(\delta_{i}=1\right)$. Following Katzman et al. (2018), we here use the negative partial log-likelihood of a Cox proportional hazards model (Cox, 1972) as a loss function for training DeepLORI:

$$l\left(W,y\right)=\sum_{i=1}^{n}\delta_{i}\left[N_{W}\left(x_{i\cdot }\right)-\log\left(\sum_{j:y_{j}\geq y_{i}}\mathrm{ex}p^{N_{W}\left(x_{j\cdot }\right)}\right)\right]$$

where $N_{W}\left(\cdot \right)$ denotes the risk score learned by DeepLORI, parameterized by weights W, and $x_{i\cdot }$ the medical history of patient i.

Given $N_{W}\left(\cdot \right)$, the hazard of a patient with feature vector *x* at time *t* is

$$h\left(t|x\right)=h_{0}\left(t\right)\mathrm{ex}p^{N_{W}\left(x\right)}$$

where $h_{0}\left(t\right)$ is the so-called baseline hazard, which can be estimated according to (Breslow, 1972):

$$h_{0}\left(t\right)=\sum_{i=1}^{n}\frac{I\left(y_{i}\leq t\right)\delta_{i}}{\sum_{j:y_{j}\geq y_{i}}\mathrm{ex}p^{N_{W}\left(x_{j\cdot }\right)}}$$

The corresponding time-dependent conditional probability for staying event-free (i.e., not suffering from the specified comorbidity) is then

$$S\left(t|x\right)=\exp\left\{-\int_{0}^{t}\mathrm{ex}p^{N_{W}\left(x\right)}\text{d}h_{0}\left(u\right)\right\}$$

To avoid over-fitting, we regularize DeepLORI during training in multiple ways:

• We use dropout units in the input and hidden layers.
• We perform batch normalization (Ioffe and Szegedy, 2015) before each activation function.
• We impose groupwise elastic net penalties for weights (Zou and Hastie, 2005).

The elastic net is an extension of the classical lasso algorithm (Tibshirani, 1996), which has originally been introduced in the context of generalized linear models. It combines an $\ell_{1}\;$penalty of coefficients (like in lasso regression) with an $\ell_{2}\;$penalty (like in ridge regression) (Hoerl and Kennard, 1970). The elastic net enforces a sparse regression model by jointly pushing coefficients toward zero via the $\ell_{1}\;$penalty, that is, there is a feature selection. At the same time, the $\ell_{2}\;$penalty promotes a joint selection of correlated features (Zou and Hastie, 2005). The idea of the elastic net can also be extended to neural networks. More specifically, by adding groupwise elastic net penalties, we modify our training objective as follows:

$$l\left(W,y\right)+\left(\sum_{s\epsilon S}\lambda_{s}^{\ell_{1}}{\left\|W_{s}\right\|}_{1}+\lambda_{s}^{\ell_{2}}{\left\|W_{s}\right\|}_{2}\right)+\lambda_{D}^{\ell_{1}}{\left\|W_{D}\right\|}_{1}+\lambda_{D}^{\ell_{2}}{\left\|W_{D}\right\|}_{2}$$

where S is the set of feature subdomains. Furthermore, $\lambda_{s}^{\ell_{1}},\lambda_{s}^{\ell_{2}}$ denote tunable hyperparameters, $W_{s}$ refers to the set of weights connecting the input to the first hidden layer within feature domain s, and $W_{D}$ are the weights of the connections feeding into the output layer.

#### Hyperparameters Optimization

A comprehensive overview of hyperparameters of DeepLORI can be found in the supplements (Supplementary Table S2). We performed Bayesian hyperparameter optimization (Bergstra et al., 2013) to tune DeepLORI on the training data. Each candidate hyperparameter set was evaluated via a 5-fold cross-validation. Hyperparameter optimization was run for 100 trials per fold, a maximum number of 100 epochs per trial, or if the cross-validated prediction performance did not increase within 10 sequential epochs. Prediction performance was measured via Harrell’s C-index (Harrell et al., 1982), which is a generalization of the area under the receiver operating characteristic curve (AUC), frequently used for classification models.

#### Shapley Additive Explanations

One of the main criticisms of neural network based approaches is the difficulty to interpret them. Recently, Lundberg and Lee (2017) proposed a model agnostic game theoretic framework to address this issue. In brief, the idea behind Shapley Additive Explanations (SHAP) is that the relevance $\Phi_{i}\left(x\right)$ of feature i on the model output f(x) can be regarded as the average weighted difference between outputs from all possible models trained on 1) all subsets *S* of features including feature i, against 2) all subsets of features excluding feature i:

$$\Phi_{i}\left(x\right)=\sum_{S\subseteq F/\left\{i\right\}}\frac{\left|S\right|!\left(\left|F\right|-\left|S\right|-1\right)!}{\left|F\right|!}\left[f_{S\cup \left\{i\right\}}\left(x_{S\cup \left\{i\right\}}\right)-\;f_{S}\left(x_{S}\right)\right]$$

with F as the set of all features. The authors propose several local approximation techniques, which can circumvent the exact combinatorial calculation of $\Phi_{i}\left(x\right),$ one which is specifically tailored toward neural networks (Deep SHAP). Deep SHAP effectively combines SHAP values calculated for smaller components of a neural network into SHAP values for the entire network. We refer to Lundberg and Lee (2017) for details. In this work, we used SHAP to understand the impact of individual feature domains, subdomains, and AEDs on the comorbidity risk score that we learned via DeepLORI. SHAP results in a patient-specific score that may be positive [feature *i* increases *f*(*x*)] or negative [feature *i* decreases *f*(*x*)] compared to the average patient. In agreement to Lundberg et al., we considered the mean absolute values of the SHAP values per feature to score the overall impact of a variable. Moreover, we investigated the overall mean absolute SHAP values per feature domain and subdomain, respectively. This is possible because SHAP values are additive. That means the sum of two SHAP values can indeed be interpreted as the overall impact of the corresponding features.

In practice, we found Deep SHAP too computationally costly when using our entire original dataset. We thus repeatedly subsampled 5% of our data with replacement (30 times) and recalculated SHAP values. We checked the robustness of the approach via the variance of SHAP values.

### Competing Methods

We compared DeepLORI against several competing approaches:

(1) Random survival forests (Ishwaran et al., 2008): In this earlier published approach (Gerlach et al., 2017), we first combined claims data with biomedical knowledge (akin to this article) and then used a window of fixed length (3°months) to summarize features via a max-pooling. Features encoding prescriptions and diagnoses within such a time window were concatenated, resulting in an overall number of around 165,000 features per patient. Subsequently, we used maximum relevance minimum redundancy (mRMR) (Ding and Peng, 2005) to further reduce the number of features to 500 prior to random survival forest (RSF) model training. For RSF model training, we relied on R-package “ranger” (Wright and Ziegler, 2017). The number of decision trees was set to 5,000, and the log-rank statistic was used as a split rule for nodes.
(2) Stacked denoising autoencoders (SDAs) followed by training an RSF (Miotto et al., 2016): In this approach, initially an SDA was trained to extract features from the medical history of each patient (diagnosis and prescription codes as well as demographic information) in an unsupervised manner. The same SDA architecture as described in Miotto et al. was employed. After feature extraction for each of the 6 comorbidities, an RSF was trained.
(3) A Kaplan–Meier (KM) estimator as “null model.” This approach does not use features of any individual. It only estimates the overall risk curve for a given comorbidity from the data and applies the same estimate to each patient. The purpose of this “null model” was to understand the added value of complex machine learning models.

### Evaluation Approach

DeepLORI was compared with competing methods within a 5-fold cross-validation scheme using the exactly same data splits of the original dataset. Hyperparameters were only tuned on the respective training data, resulting in a nested cross-validation scheme for DeepLORI. We used Uno’s C-index (Uno et al., 2011) as a performance measure:

$${\hat{C}}_{\tau }=\frac{\sum_{i=1}^{n}\sum_{j=1}^{n}\Delta_{i}{\left\{\hat{G}(X_{i}\right\}}^{-2}I\left(X_{i}<X_{j},X_{i}<\tau \right)I\left({\hat{risk}}_{i}>{\hat{risk}}_{i}\right)}{\sum_{i=1}^{n}\sum_{j=1}^{n}\Delta_{i}{\left\{\hat{G}(X_{i}\right\}}^{-2}I\left(X_{i}<X_{j},X_{i}<\tau \right)}$$

Uno’s C-index is a consistent estimator of the concordance index for a population that is independent of censoring. It satisfies this requirement for censored populations using two “tricks,” first by applying an “inverse probability weighting” schema using the censoring distribution estimated with $\hat{G}\left(\cdot \right)$ (e.g., the Kaplan–Meier estimator), second by evading instable tail parts for times ≥τ of the estimated survival function with a prespecified time point τ as constraint. We refer to Uno et al. (2011) for further details.

In addition, we evaluated DeepLORI on our external validation data. This validation was done separately in two different ways:

(1) Follow-up of existing patients: We selected patients who had already been in our original dataset, but for whom a right censoring was observed.
(2) New patients: We evaluated DeepLORI on patients who had no records in the original dataset.

In both cases, we recorded Uno’s C-index over the entire time series and as a function of time (named AUC(t) in the following) to measure the prediction performance.

## Results

### DeepLORI Outperforms Competing Methods

**FIGURE 3:**
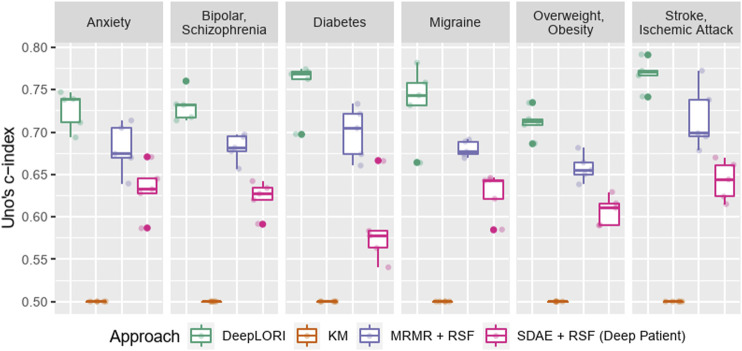
Five-fold (nested) cross-validation test sets performance benchmark of Deep LORI (green) vs. competing methods; mRMR: minimum redundancy maximum relevance feature selection; SDAE: stacked denoising autoencoder; RSF: random survival forest; KM: Kaplan–Meier estimator.

Despite high censoring rates, all 6 comorbidities could be predicted rather accurately by DeepLORI, and the 5-fold cross-validated performance of Uno’s C-index ranged from 71% for overweight and obesity to 77% for stroke and ischemic attacks (Figure 3; Supplementary Table S3). At the same time, the Kaplan–Meier estimator (i.e., the “null”-model without any features) was consistently at the chance level (50% Uno’s C-index), indicating that all of our tested machine learning models (DeepPatient, DeepLORI, and MRMR + RSF) extracted relevant predictive signal from the data. At the same time, DeepLORI showed significantly higher C-indices than all competing methods.

### DeepLORI Shows Stable Prediction Performance on External Validation Data

**FIGURE 4:**
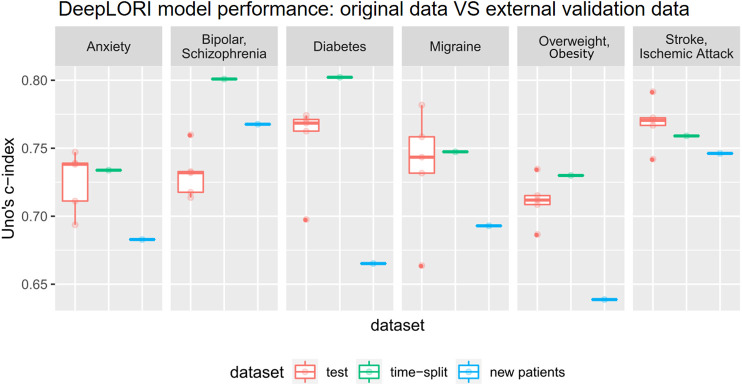
Prediction performance of Deep LORI on external validation data. Green: time-split validation, that is, follow-up of patients included in training data. Blue: prediction performance on new patient data. Red: 5-fold cross-validated prediction performance on original data for comparison purposes.

Evaluation of DeepLORI on the external validation data showed roughly comparable C-indices to those observed on the original data when focusing on the follow-up of the ∼15,000 patients, who had medical history in the original data (Figure 4). This highlights that DeepLORI, despite high censoring rates in the original data, was not over-fitted. C-indices for new/so far unseen patients (*n* = ∼97,000) in the external validation data were around 6% lower (Supplementary Table S3).

**FIGURE 5:**
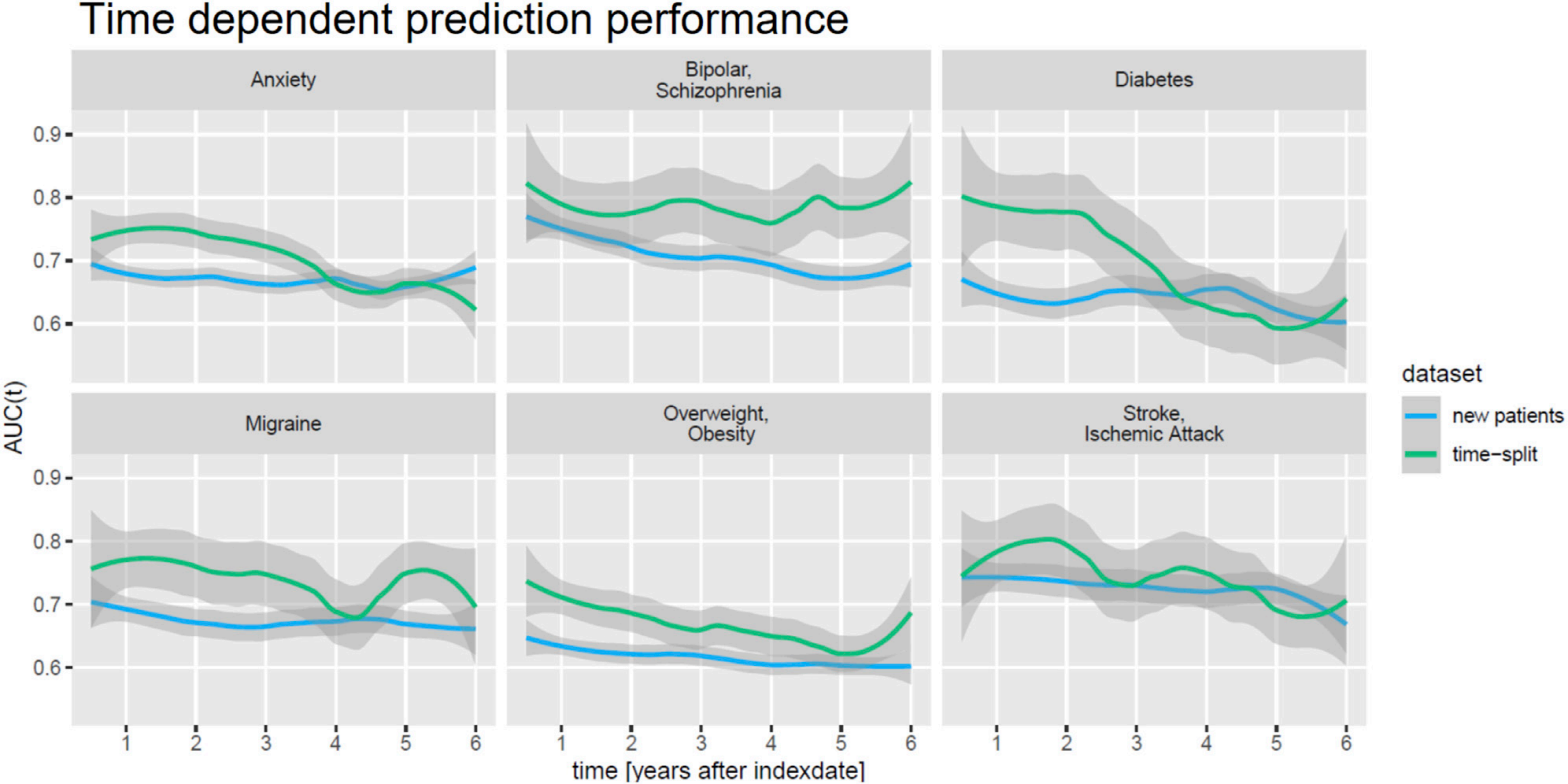
Time-dependent prediction performance (AUC(t)) of DeepLORI on external validation data.

When investigating the AUC(t), we found a rather stable prediction performance for all comorbidities over time (Figure 5). Remarkably, this held true for a time interval of up to 6°years after initial diagnosis of epilepsy, and it was true for the follow-up of existing patients as well as for new patients, again highlighting the fact that DeepLORI generalizes rather well.

### DeepLORI Models Are Explainable

**FIGURE 6:**
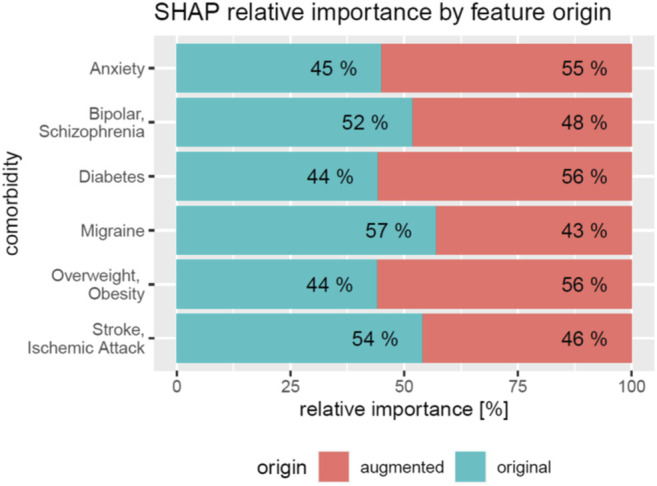
Relative impact of original vs augmented features in DeepLORI models for 6 different comorbidities

**FIGURE 7:**
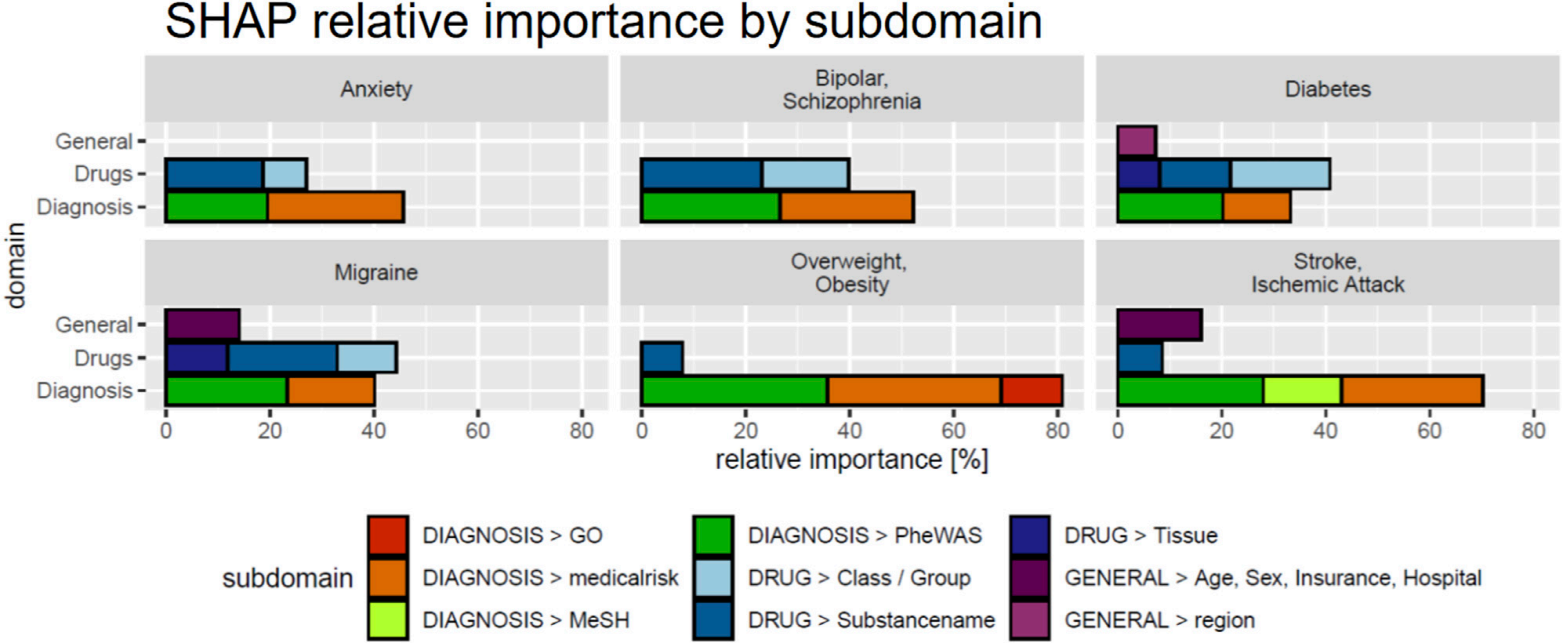
SHAP values by feature subdomain. Subdomains with a cumulative relative importance ≤5% are not shown.

We next investigated the relevance of feature domains via SHAP in DeepLORI models trained on the entire original dataset. This analysis highlighted for most comorbidities the relevance of features derived via augmentation of the original data with additional information (Figure 6): For example, in the model, predicting anxiety augmented features made up around 55% of the total feature importance, and for migraine, we found 43%. Notably, among augmented features, the “medical risk” subdomain, covering various unspecific comorbidity indices derived from ICD diagnosis codes (Charlson et al., 1987; Romano et al., 1993; Lee et al., 1999; Schneeweiss et al., 2003; Boersma et al., 2005; Quan et al., 2005; Sessler et al., 2010; Sigakis et al., 2013) using the R-package “medicalrisk” (McCormick and Joseph, 2020) in the medical history of epilepsy patients, had a significant impact (Figure 7), suggesting that the risk of developing any of our six comorbidities increases with a generally worse medical condition upfront. Importantly, none of the comorbidity indices are specific to any of the six comorbidities focused by DeepLORI.

**FIGURE 8:**
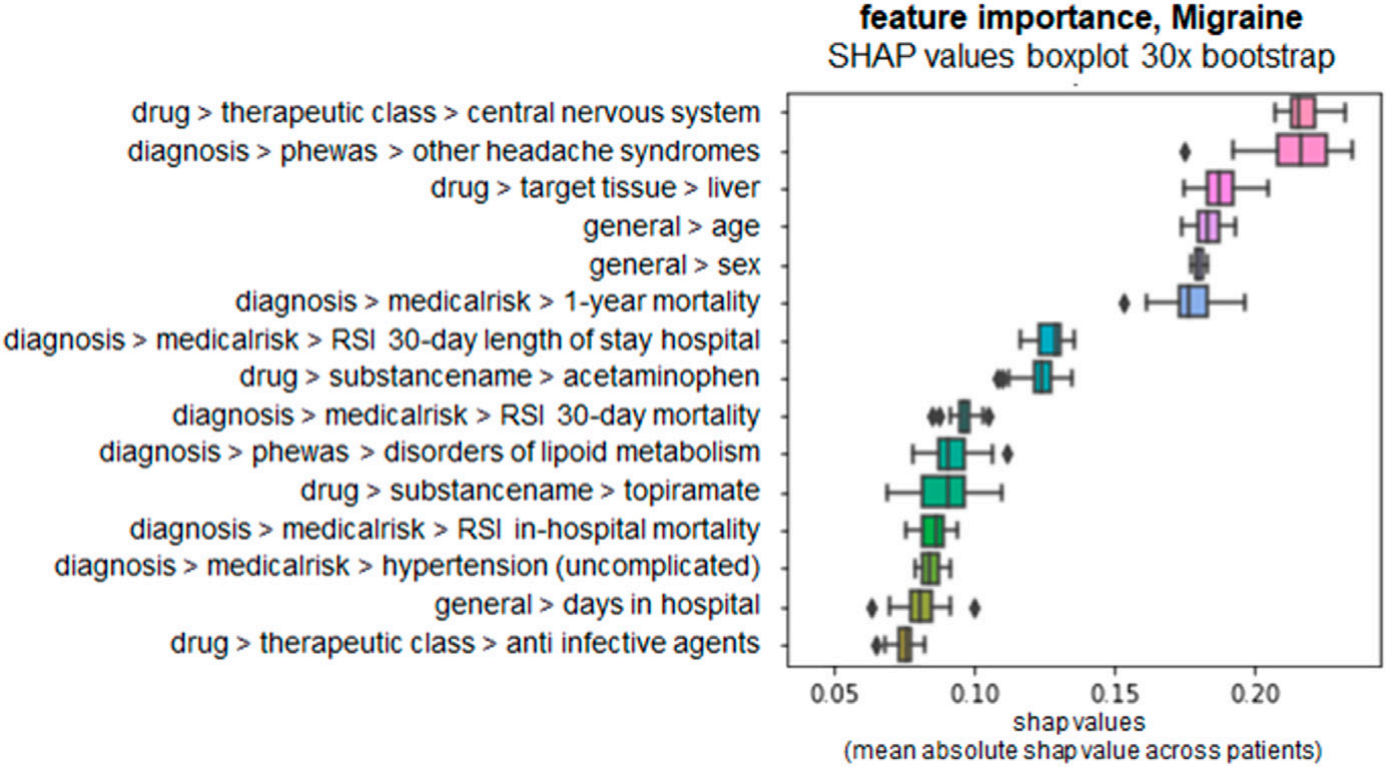
SHAP values of top 15 most relevant features in the DeepLORI model for migraine, rank by mean absolute values. *x*-axis: SHAP values, higher values = higher importance. *y*-axis: features ranked by importance, annotated with their domain and subdomain membership (domain > subdomain > feature).

**FIGURE 9:**
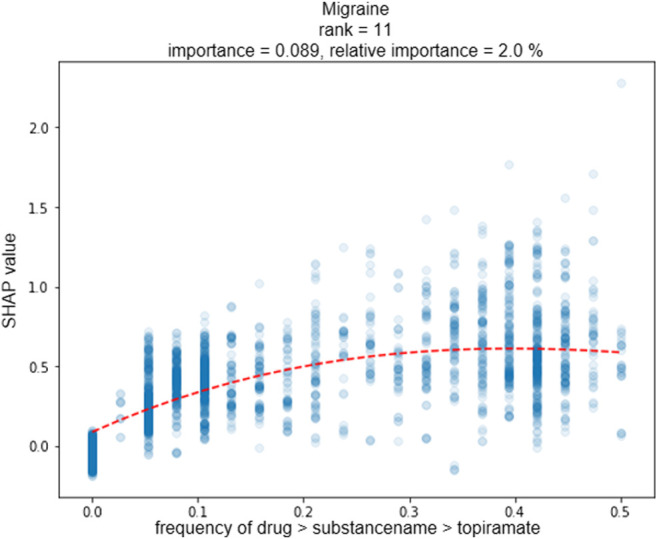
Plot shows the marginal dependency of the frequency of topiramate prescription on the predicted risk on later migraine diagnosis. One dot represents one patient; *x*-axis: prescription frequency (0% = never observed, 100% = observed every month of a patient‘s medical history); *y*-axis: change in SHAP value (=change in individual hazard rate). Marginal dependency plots for each comorbidity for the top 5 features can be found in the Supplemental Material (Supplementary Figures S7–S12).

In the following, we discuss one of the six DeepLORI models in more detail, namely, the one for migraine (Figure 8). (Figures related to DeepLORI models for the other five comorbidities can be found in the Supplementary Material (Supplementary Figures S1–S6)). According to SHAP analysis, the most relevant features in the DeepLORI model for migraine relate to the prior existence of headaches and the use of drugs for the nervous system, which are typically used to treat headaches. In addition, many drugs used for treating headaches are known to affect the liver (Mathew and Klein, 2019; Valade, 2019). It is known that females are more affected by migraine than males, and that migraine is age-dependent (Victor et al., 2010). The antiepileptic drug (AED) topiramate, known to be well-tolerated by this group of patients (Spritzer et al., 2016; Silberstein, 2017), ranks among the top 15 most relevant features. Figure 9 shows the marginal dependency of DeepLORI model predictions on AED prescription frequency, suggesting that patients treated with topiramate are slightly more likely to be diagnosed with migraine later than those without such treatment in the past. In fact, topiramate is often used as a preventive treatment for migraine (Spritzer et al., 2016), suggesting that patients treated with this AED are often considered at risk of developing migraine by their treating physician. Indeed, many of these patients eventually receive this diagnosis.

Another interesting finding from the SHAP analysis of our model is the influence of disorders of the lipoid metabolism on migraine risk. Associations between lipid levels and migraine have been reported in Rist et al. (2011) and Onderwater et al. (2019). Moreover, the metabolic syndrome and migraine have been associated with each other (Sachdev and Marmura, 2012). It is important to highlight at this point that SHAP analysis does not provide a causal explanation, though.

**FIGURE 10:**
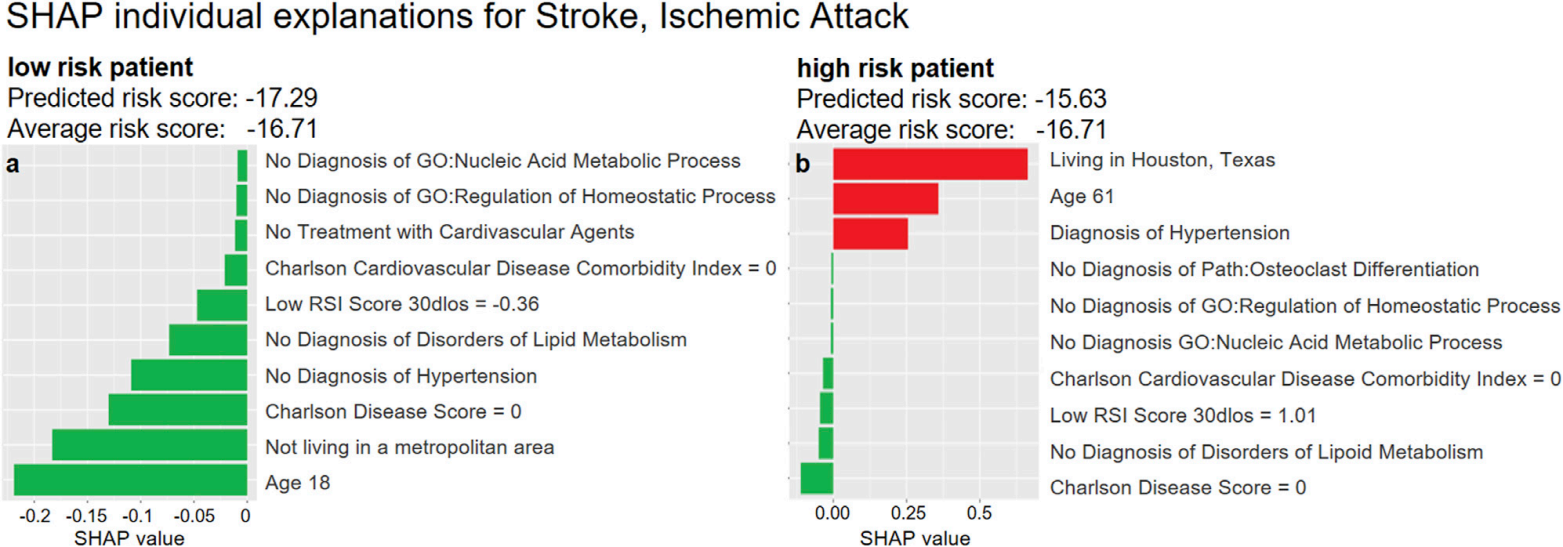
Examples of SHAP explanations for a patient predicted at low risk for stroke and ischemic attacks **(left)** and a high-risk patient **(right)**. Red (green) bars indicate higher **(lower)** risk than the average patient: The hazard ratio^2^ of the low-risk patient **(left)** is around 56% of that of an average patient (exp(−17.29)/exp(−16.71) =0.56). The hazard ratio of the high-risk patient **(right)** is around 2.94 times that of an average patient ( exp(−15.63)/exp(−16.71)=2.94).

To further exemplify the possibility of interpreting our DeepLORI models on the level of individual patients, we depict in Figure 10 SHAP values for two randomly selected patients with high and low risk for developing stroke or ischemic attacks, respectively. As expected, the low-risk patient is young and has no diagnosis of hypertension or disorder of the metabolic system. In contrast, the high-risk patient is an older person with hypertension who lives in Texas. In fact, significant regional differences in the risk for strokes have been reported throughout the United States (Howard et al., 2007), and Eastern Texas belongs to the so-called “Stroke Belt” (Karp David et al., 2016).

## Discussion and Conclusion

Precision medicine has the vision to bring the right treatment to the right patients. Precision medicine is strongly dependent on machine learning. At present, precision medicine is only an emerging reality. Several reasons can be identified (Fröhlich et al., 2018; Miotto et al., 2018; Xiao et al., 2018; Kwak and Hui, 2020): 1) lack of the right data in sufficient quantity, 2) insufficient validation, and 3) difficulties in interpreting complex ML models, which is by itself a prerequisite for generating the necessary confidence in using such models. Realization of precision medicine will only be possible, if all these aspects are addressed jointly. In this context, it is essential that ML models can be used in a cost-effective and practical manner. Hence, clinical routine data are of extreme relevance and are gaining more and more attention (Weiss et al., 2012; Peissig et al., 2014; Choi et al., 2016; Miotto et al., 2016; Rajkomar et al., 2018; Harutyunyan et al., 2019). Administrative claims data constitute an important source of such clinical routine data. They principally exist in large quantities and allow for obtaining insights into the longitudinal medical history of individual patients under real-world conditions. However, these data have not been collected for research purposes. First of all, coding of diagnoses into ICD codes is not unique and mostly done for maximizing economic reasons, rather than for providing a precise medical description. Different ICD codes can be used for similar diagnoses, and the relationship among different medical conditions is consequently not always uniquely resolvable from their distances in the ICD ontology. Second, it should be noted that ICD only reflects the medical symptom level, which should not be confused with the biological relationship among disorders. Third, the time of diagnosis encoding might not correspond to the actual appearance of the medical condition. Fourth, it is unclear whether patients take the prescribed medication. Finally, the nature of irregular time series data, different for each patient, imposes specific challenges for data analysis.

In this work, we tried to address these challenges by a) mapping ICD codes to PheWAS codes that are at higher granularity, b) augmenting the original data with further information from biological databases, and c) proposing specific multimodal neural network architecture (DeepLORI). We demonstrated that DeepLORI can predict six common comorbidities of epilepsy patients with higher C-index than several competing methods. We performed a rigorous cross-validation plus an external validation to assess our model, demonstrating that DeepLORI allows for reliable predictions of comorbidity risks up to six years in advance. We showed that with the help of SHAP and our data augmentation approach, it is possible to make DeepLORI-based predictions explainable, even on the level of individual patients. From our perspective, this is of great importance for generating confidence in ML-based solutions in medicine.

From a medical perspective, we see the value of our work in the potential for much earlier identification of epilepsy patients at risk of developing different comorbidities. For example, a patient at high risk of developing diabetes type 2 should consider losing weight and regularly check insulin levels. A patient at high risk of developing psychiatric disorders might consider early consultation with a psychiatrist. Hence, risk models could be a way to eventually move toward preventive medicine.

Further applications of our work could lie in addressing the high subject-to-subject variability in epilepsy: Based on the comorbidity risk profile learned by DeepLORI, one might be able to identify subgroups of patients with more homogenous disease progression, potentially opening up opportunities for developing more personalized therapies in the future.

## Data Availability

The data analyzed in this study are subject to the following licenses/restrictions: We used administrative health claims data provided by IBM Truven Health Analytics. Data access has to be requested from IBM. The DeepLORI framework is now available at https://gitlab.scai.fraunhofer.de/thomas.linden/deeplori. The readme contains instructions for a setup and demo. The demo simulates patients who are accessible from the described folder. Requests to access these datasets should be directed to https://www.ibm.com/products/marketscan-research-databases, https://www.ibm.com/watson-health/about/truven-health-analytics.

## Footnotes

1
http://www.tdrdata.com/ipd/ipd ICD10ToICD9List

2 Hazard ratios computed similar to Cox proportional hazard's model (Cox, 1972).

## Abbreviations

AED, antiepileptic drug; SDA, stacked denoising autoencoder; mRMR, minimum redundancy maximum relevance; RSF, random survival forest.
