# Supplementary material for: An Explainable Multimodal Neural Network Architecture for Predicting Epilepsy Comorbidities Based on Administrative Claims Data
Source: Front Artif Intell. 2021 May 21;4:610197. doi: 10.3389/frai.2021.610197 (PMC8176093; doi:10.3389/frai.2021.610197)
Supplement: Supplementary file 1 [file DataSheet1.docx]

**Supplementary Material**
Deep LORI: An Explainable Multi-Modal Neural Network Architecture for Predicting Epilepsy Comorbidities Based on Administrative Claims Data
Thomas Linden [née Gerlach^[[1]](#footnote-1)^]^a,b,c^ , Johann de Jong^c^, Chao Lu^d^, Victor Kiri^d^, Kathrin Haeffs^e^, Holger Fröhlich^a,b,c^

^a^Department of Bioinformatics, Fraunhofer Institute for Algorithms and Scientific Computing (SCAI),
Schloss Birlinghoven, Sankt Augustin, Germany

^b^Bonn-Aachen International Center for Information Technology (B-IT), University of Bonn, Bonn, Germany

^c^UCB Biosciences GmbH, Monheim, Germany

^d^UCB Ltd., Raleigh, USA

# Data

## Claims Based Electronic Health Records

We were using two cohorts:

1. The „original data“ which was queried from the IBM MarketScan databases covering the years 2011-2015 (5 years of data): The raw unfiltered data had 535,492 patients and after applying the filter criteria 19,517 patients remain;
2. The „external validation data“ covering the years 2008-2018 (11 years of data) of raw size of 1,283,303 patients and after applying the same criteria as for the training dataset 112,755 patients remain. Hence there is no increase through filtering, instead 2 datasets of different size were queried using the same filter criteria, leading to 19,510 and 112,755 patients, respectively.

## Definition of Focused Comorbidities and Compilation of Training Data

| Comorbidity | PheWAS Code description |
| --- | --- |
| Anxiety | "Anxiety disorder",  "Generalized anxiety disorder",  "Anxiety, phobic and dissociative disorders", "Agorophobia, social phobia, and panic disorder" |
| Bipolar, Schizophrenia | "Bipolar",  "Schizophrenia and other psychotic disorders" |
| Diabetes | "Type 2 diabetes" |
| Migraine | "Migraine",  "Migrain with aura" |
| Overweight, Obesity | "Overweight", "Obesity" |
| Stroke, IschemAttack | "Ischemic stroke",  "Transient cerebral ischemia" |

**Table S 1:** Overview of comorbidity defintions using PheWAS.

# Methods

### Integration of Biological Background Knowledge into Claims Data

We used DisGeNET (Piñero *et al.*, 2017) to retrieve for each reported diagnosis in our data disease associated genes. Enrichment of Gene Ontology (GO) biological processes (‘The Gene Ontology Consortium’, 2004), KEGG (Kanehisa, 2000) and Wiki pathways (Slenter *et al.*, 2018) was then estimated via a conditional hyper-geometric test using GOstats (Falcon and Gentleman, 2007) with a tail area based false discovery rate (Strimmer, 2008) cutoff of 5% for GO and 20% for pathways. Furthermore, known disease biomarkers and symptoms were obtained from the Therapeutic Target Database (TTD) (Yang *et al.*, 2016) and the human symptoms-disease network (Zhou *et al.*, 2014). Similarly, medications in the claims data were mapped to known targets via TTD and DrugBank (Wishart, 2006) via text matching of substance names, and information about tissue expression of drug targets was obtained from the Human Protein Atlas (Uhlen *et al.*, 2015). Potential side effects of drugs and their likelihoods were retrieved from SIDER (Kuhn *et al.*, 2016), again by application of text matching of substance names. We obtained information about likely indication areas of drugs from MEDI (Wei *et al.*, 2013). Notably, only indication areas that were marked as “high confidence” by the MEDI authors and were mentioned in at least 10 PubMed articles were used. Finally, medications were mapped to substance classes and groups via the RED BOOK™ database. Further details are described in (Gerlach, Lu and Fröhlich, 2017).

### Hyperparameters Optimization

| **hyperparameter** | **sample space** | **lower  bound** | **upper  bound** | **choice** | **comment** |
| --- | --- | --- | --- | --- | --- |
| architecture | | | | | |
| #layers | choice |  |  | {1, 2, 3, 4} |  |
| #units/layer shrinkage | conditional on  #layers |  |  | 1 layer: {1%, 0.1%} 2-4 layers: {10%, 1%} |  |
| activation function | choice |  |  | tanh, selu |  |
| initializer | condtitional on  activation function |  |  | tanh: {glorot normal, orthogonal} selu: {lecun normal} |  |
| pooling method | choice |  |  | {max, mean} | 3 windows: [3,6,17] |
| convolution | choice |  |  | {yes, no} | 5 filters per window. 3 windows: [3,6,17] |
| regularization | | | | | |
| L1 | log_uniform | 1.00E-02 | 1.00E-07 |  |  |
| L2 | log_uniform | 1.00E-02 | 1.00E-07 |  |  |
| dropout input | uniform | 0% | 10% |  |  |
| dropout hidden | uniform | 0% | 50% |  |  |
| batch normalization | choice |  |  | {yes, no} | allow "no" for selu |
| batch size | choice |  |  | {64,128,256,512} | (Keskar *et al.*, 2016) |
| training configuration | | | | | |
| learning rate | log_uniform | 1.00E-01 | 1.00E-04 |  |  |
| optimizer | choice |  |  | {SGD nesterov, RMSProp, Nadam} |  |

**Table S 2:** Overview of hyperparameters tuned with Bayesian Hyperparameter Optimization. Hyperparameters were sample from continuous distributions (uniform, log-uniform), discrete (#layers, #units/layer, batch size) and categorical (all others) spaces. Some subspaces are conditional on the sampling outcome of another hyperparameter, e. g. the initializer depends on the activation function and the width (#units/layer shrinkage) depends on the depth (#layers).

### Details on Modeling Time Dependency

In our case max pooling corresponds to a mapping from $\left\{ 0,1 \right\}^{w}\to\left\{ 0,1 \right\}$performing a qualitative aggregation and mean pooling from $\left\{ 0,1 \right\}^{w}\to[0,1]$to perform a quantitative aggregation across a patients medical history. Where $w$ are the number of timepoints per feature for a given window size, e.g. $w=3$ means that data from 3 consectuive timpoints are aggregated.

## DeepLORI Outperforms Competing Methods

|  | Uno's c-index | | | |
| --- | --- | --- | --- | --- |
|  | original data | external validation data (n=112,755) | | Difference  time-split VS new patients |
| comorbidity | test set (n=19,510) | time-split (n=15,276) | new patients (n=97,479) |  |
| Anxiety | 0.73 (0.02) | 0.73 | 0.68 | 0.05 |
| Bipolar, Schizophrenia | 0.73 (0.02) | 0.80 | 0.77 | 0.03 |
| Diabetes | 0.75 (0.03) | 0.80 | 0.67 | 0.13 |
| Migraine | 0.74 (0.04) | 0.75 | 0.69 | 0.06 |
| Overweight, Obesity | 0.71 (0.02) | 0.73 | 0.64 | 0.09 |
| Stroke, Ischemic Attack | 0.77 (0.02) | 0.76 | 0.75 | 0.01 |

**Table S3:**  Prediction performances (Uno’s c-index) of the observed endpoints. Original data: 5x5-fold nested cross-validation; mean = mean performance of the best model; *se = standard error of the mean. External validation data: Prediction performance based on the final models.

## Interpretation of DeepLORI Models via SHAP

## Boxplot 30x bootstrap top-15 features

The boxplots in this section show the distribution of shap values: We repeatedly subsampled 5% of our data with replacement (30 times) and re-calculated SHAP values. We checked the robustness of the approach via the variance of SHAP values.


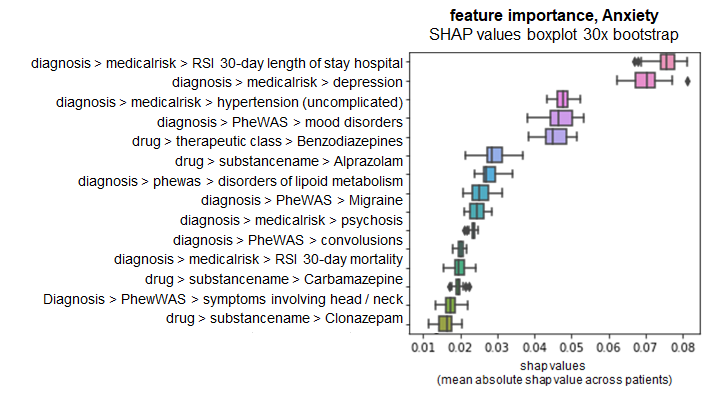


**Figure S 1:** SHAP boxplot feature importance Anxiety


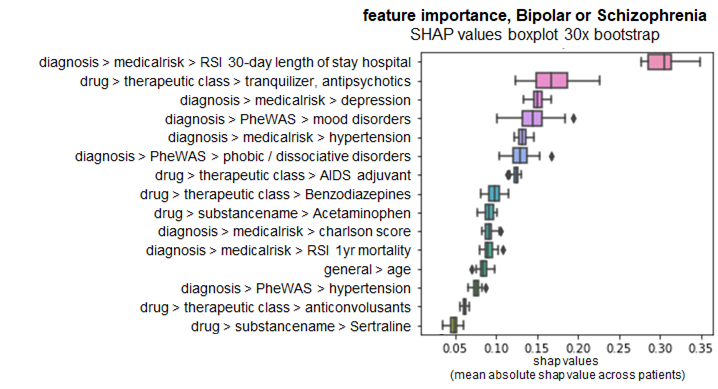


**Figure S 2:** SHAP boxplot feature importance Bipolar or Schizophrenia


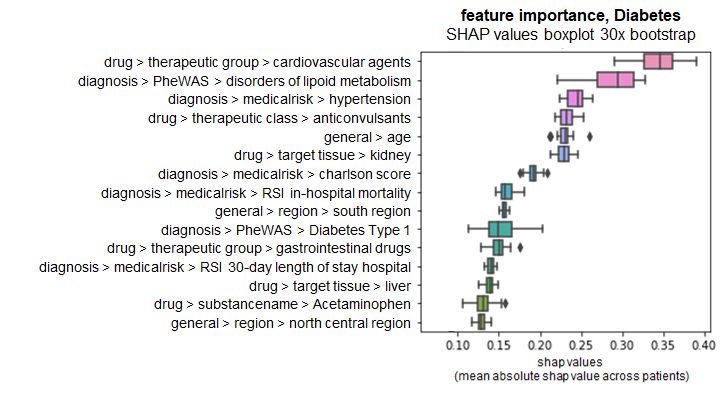


**Figure S 3:** SHAP boxplot feature importance Diabetes type 2


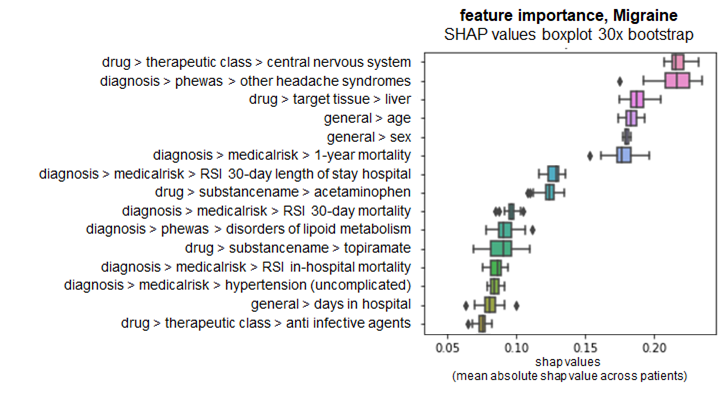


**figure S 4:** SHAP boxplot feature importance Migraine


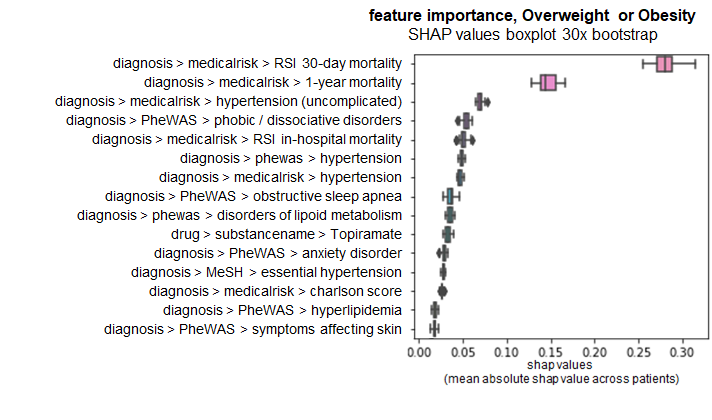


**figure S 5:** SHAP boxplot feature importance Overweight or Obesity


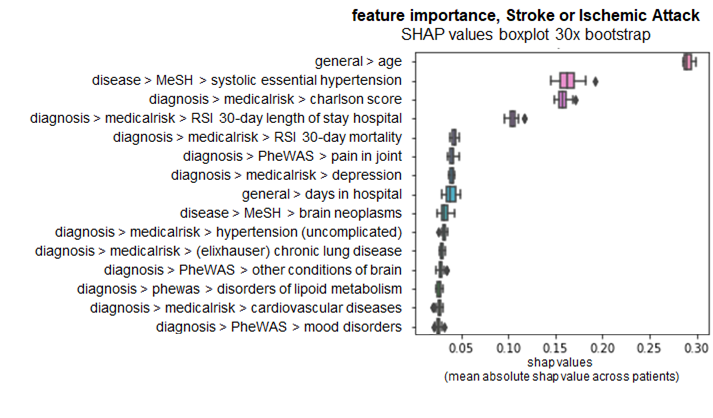


**figure S 6:** SHAP boxplot feature importance Stroke or Ischemic Attack

### Marginal dependency plots for top 5 features

The following plots show marginal influences of the top 5 features (according to the mean absolute SHAP value, see before) on predicted comorbidity risks.

- x-axis denotes feature values
- y-axis denotes the SHAP value (higher value = higher influence on predicted comorbidity risk (hazard rate) compared to average patient)

#### Anxiety


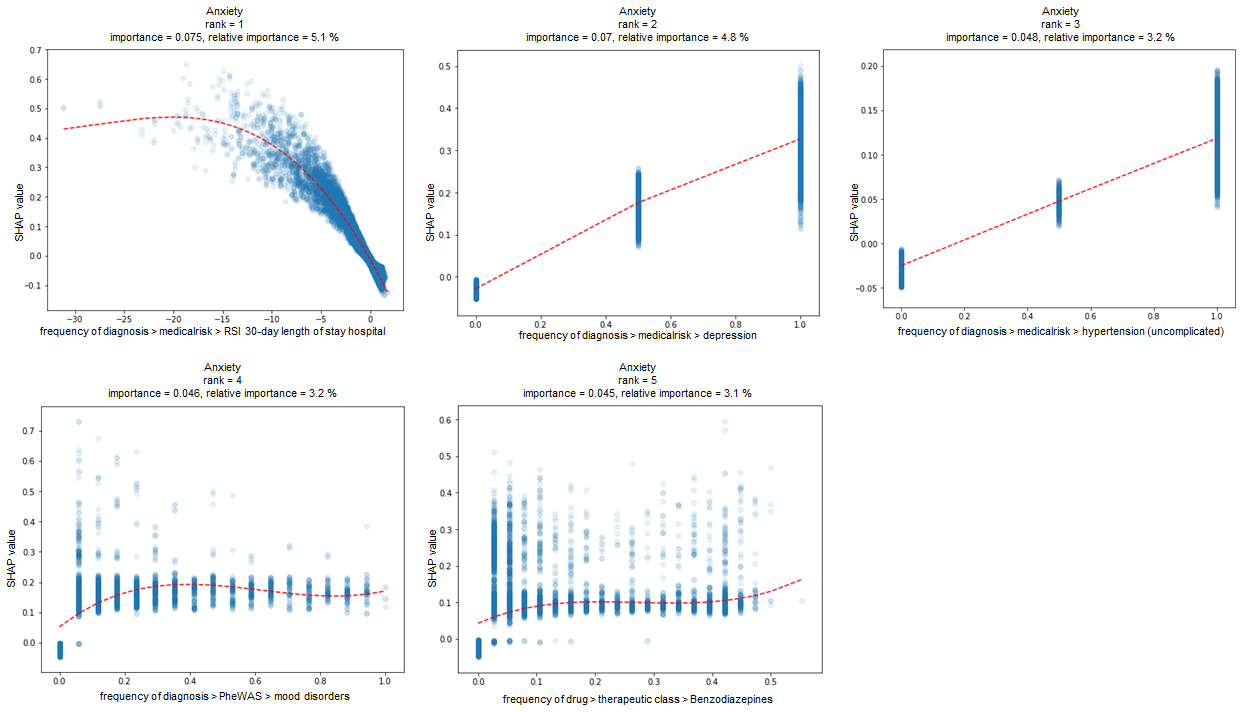


**Figure S 7:** SHAP marginal dependency plot Anxiety

#### Bipolar, Schizophrenia


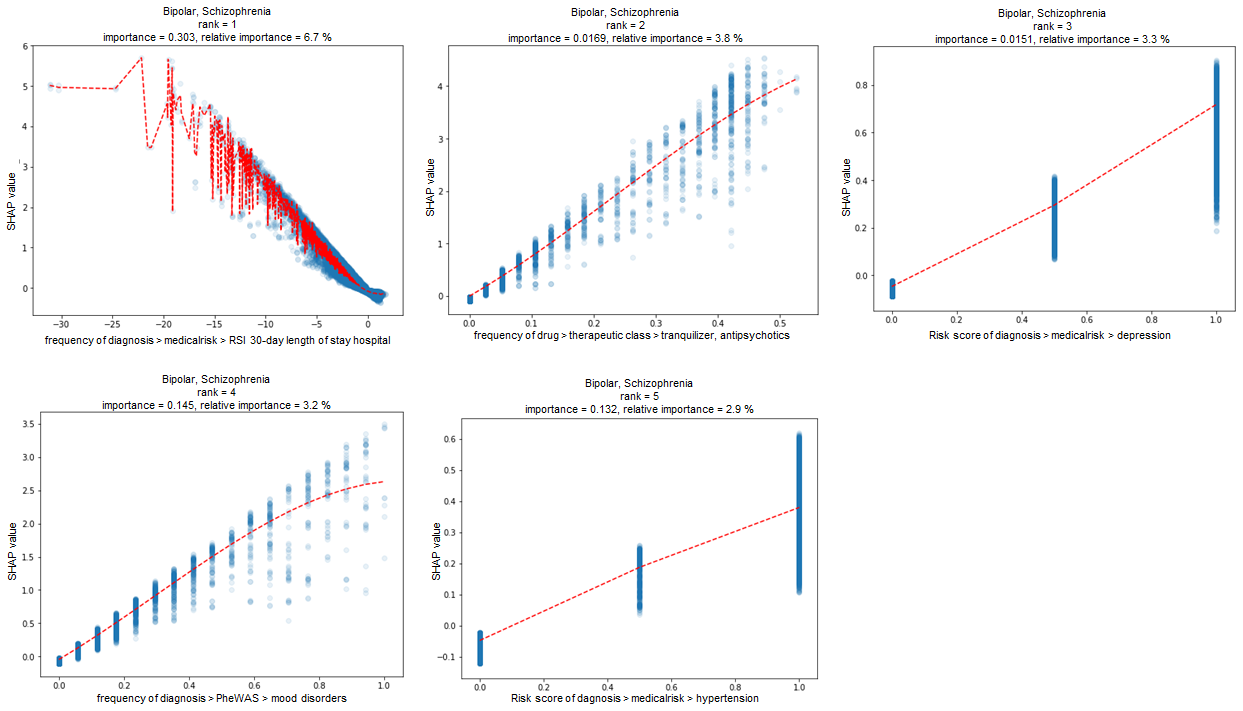


**Figure S 8:** SHAP marginal dependency plot Bipolar, Schizophrenia

#### Diabetes type 2


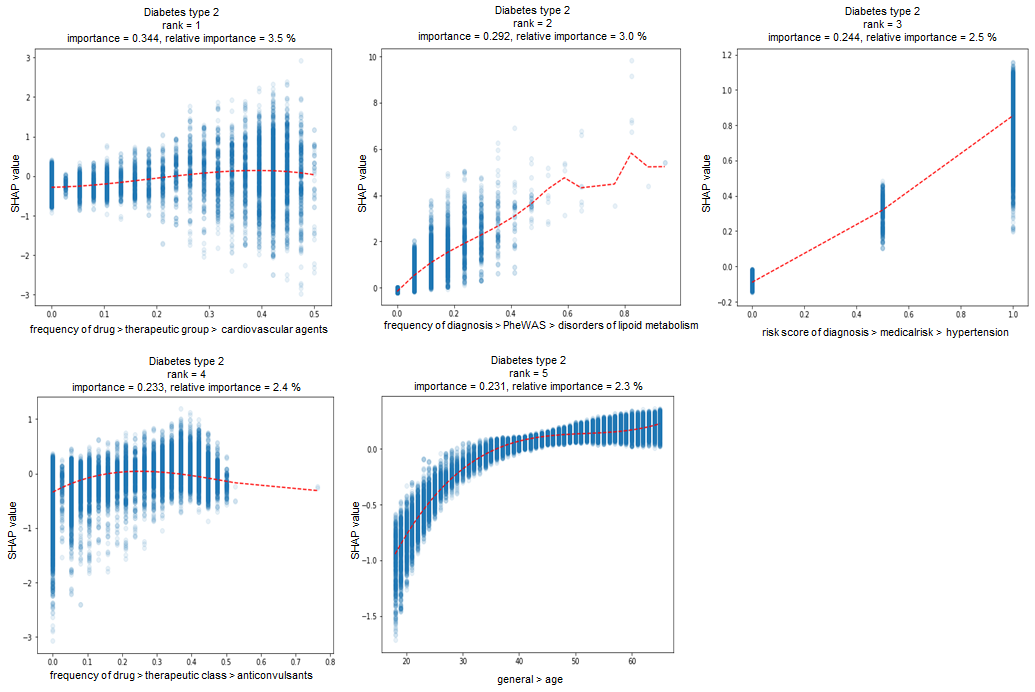


**Figure S 9:** SHAP marginal dependency plot Diabetes type 2

#### Migraine


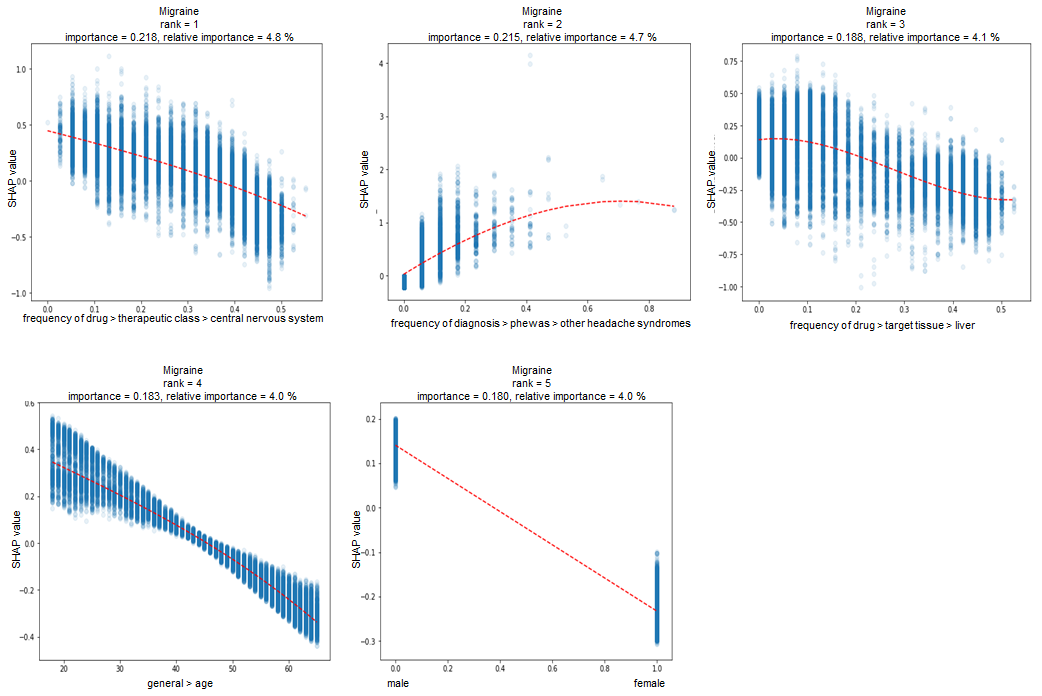


**Figure S 10:** SHAP marginal dependency plot Migraine

#### Overweight, Obesity


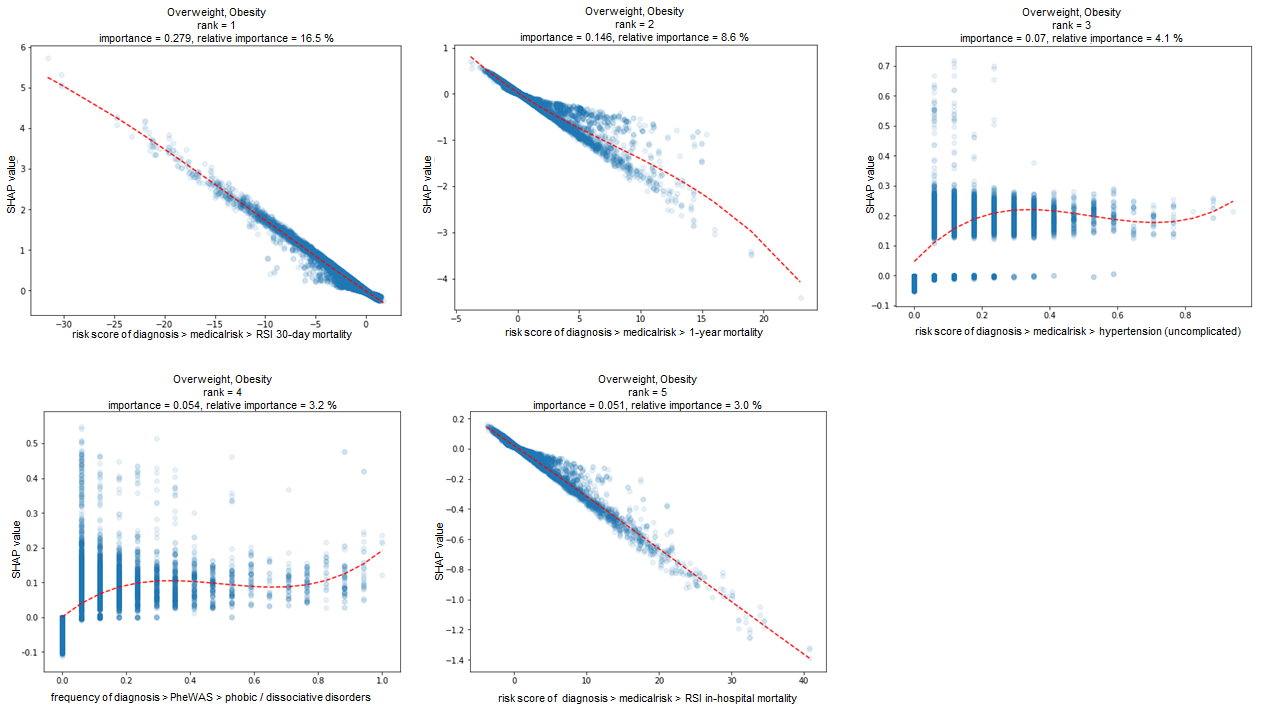


**figure S 11:** SHAP marginal dependency plot Overweight, Obesity

#### Stroke, Ischemic Attack


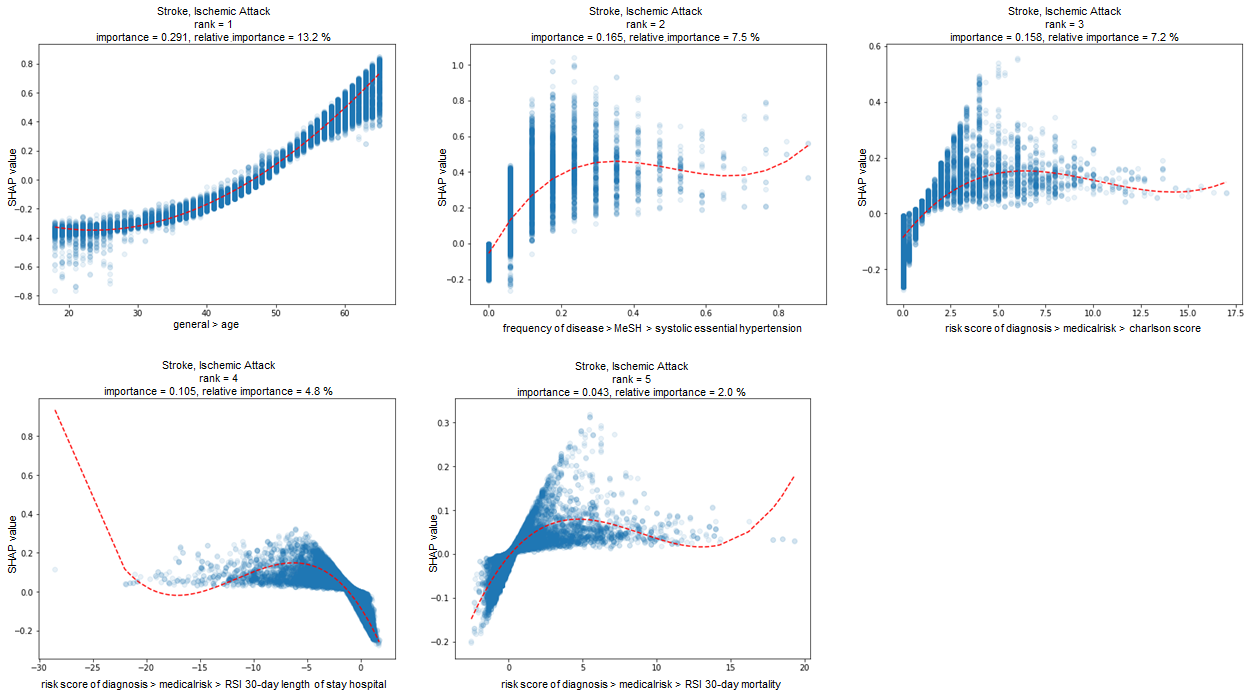


**figure S 12:** SHAP marginal dependency plot Stroke, Ischemic Attack

**References**

Falcon, S. and Gentleman, R. (2007) ‘Using GOstats to test gene lists for GO term association’, *Bioinformatics (Oxford, England)*, 23(2), pp. 257–258. doi: 10.1093/bioinformatics/btl567.

Gerlach, T., Lu, C. and Fröhlich, H. (2017) ‘Predicting comorbidities of epilepsy patients using big data from Electronic Health Records combined with biomedical knowledge’, in *Proceedings of the GCB German Conference on Bioinformatics 2017*. *GCB 2017*, Tübingen, Germany: PeerJ Inc. doi: 10.7287/peerj.preprints.3228v1.

Kanehisa, M. (2000) ‘KEGG: Kyoto Encyclopedia of Genes and Genomes’, *Nucleic Acids Research*, 28(1), pp. 27–30. doi: 10.1093/nar/28.1.27.

Keskar, N. S. *et al.* (2016) ‘On Large-Batch Training for Deep Learning: Generalization Gap and Sharp Minima’, *arXiv:1609.04836 [cs, math]*. Available at: http://arxiv.org/abs/1609.04836 (Accessed: 29 August 2018).

Kuhn, M. *et al.* (2016) ‘The SIDER database of drugs and side effects’, *Nucleic Acids Research*, 44(Database issue), p. D1075. doi: 10.1093/nar/gkv1075.

Piñero, J. *et al.* (2017) ‘DisGeNET: a comprehensive platform integrating information on human disease-associated genes and variants’, *Nucleic Acids Research*, 45(D1), pp. D833–D839. doi: 10.1093/nar/gkw943.

Slenter, D. N. *et al.* (2018) ‘WikiPathways: a multifaceted pathway database bridging metabolomics to other omics research’, *Nucleic Acids Research*, 46(D1), pp. D661–D667. doi: 10.1093/nar/gkx1064.

Strimmer, K. (2008) ‘A unified approach to false discovery rate estimation’, *BMC Bioinformatics*, 9(1), p. 303. doi: 10.1186/1471-2105-9-303.

‘The Gene Ontology Consortium’ (2004) *Nucleic Acids Research*, 32(Database issue), pp. D258–D261. doi: 10.1093/nar/gkh036.

Uhlen, M. *et al.* (2015) ‘Tissue-based map of the human proteome’, *Science*, 347(6220), pp. 1260419–1260419. doi: 10.1126/science.1260419.

Wei, W.-Q. *et al.* (2013) ‘Development and evaluation of an ensemble resource linking medications to their indications’, *Journal of the American Medical Informatics Association*, 20(5), pp. 954–961. doi: 10.1136/amiajnl-2012-001431.

Wishart, D. S. (2006) ‘DrugBank: a comprehensive resource for in silico drug discovery and exploration’, *Nucleic Acids Research*, 34(90001), pp. D668–D672. doi: 10.1093/nar/gkj067.

Yang, H. *et al.* (2016) ‘Therapeutic target database update 2016: enriched resource for bench to clinical drug target and targeted pathway information’, *Nucleic Acids Research*, 44(D1), pp. D1069-1074. doi: 10.1093/nar/gkv1230.

Zhou, X. *et al.* (2014) ‘Human symptoms–disease network’, *Nature Communications*, 5(1), p. 4212. doi: 10.1038/ncomms5212.

1. Last name changed from Gerlach to Linden in 2018 [↑](#footnote-ref-1)
